# Supplementary material for: Distribution and treatment needs of soil-transmitted helminthiasis in Bangladesh: A Bayesian geostatistical analysis of 2017-2020 national survey data
Source: PLoS Negl Trop Dis. 2023 Nov 6;17(11):e0011656. doi: 10.1371/journal.pntd.0011656 (PMC10662736; doi:10.1371/journal.pntd.0011656)
Supplement: S1 Appendix — (PDF) [file pntd.0011656.s001.pdf]

S1 Appendix: Covariates overview

Table 1 gives an overview over the used covariates including their sources and spatial and temporal resolutions. Figures 1 and 2 display the geographical distribution of selected variables based on the bivariate analysis and include an overview over the 64 districts in Bangladesh.

Table 1: Description, source, time period of collection and spatial and time resolution of the covariates included in the analysis. If the period of collection contained several measurements, the data was aggregated over the indicated time period.

Description	Source	Period	Resolution
Annual Mean Temperature	MODIS	2015-2019	1x1km^2, 8 days
Mean Diurnal Range (Mean of monthly (max temp - min temp))	MODIS	2015-2019	1x1km^2, 8 days
Isothermality (BIO2/BIO7) ($\times$100)	MODIS	2015-2019	1x1km^2, 8 days
Temperature Seasonality (standard deviation $\times$100)	MODIS	2015-2019	1x1km^2, 8 days
Max Temperature of Warmest Month	MODIS	2015-2019	1x1km^2, 8 days
Min Temperature of Coldest Month	MODIS	2015-2019	1x1km^2, 8 days
Temperature Annual Range (BIO5-BIO6)	MODIS	2015-2019	1x1km^2, 8 days
Mean Temperature of Wettest Quarter	MODIS/CHIRPS	2015-2019	1x1km^2/5.6x5.6km^2, 8 days/5 days
Mean Temperature of Driest Quarter	MODIS/CHIRPS	2015-2019	1x1km^2/5.6x5.6km^2, 8 days/5 days
Mean Temperature of Warmest Quarter	MODIS	2015-2019	1x1km^2, 8 days
Mean Temperature of Coldest Quarter	MODIS	2015-2019	1x1km^2, 8 days
Annual Precipitation	CHIRPS	2015-2019	5.4x5.4km^2, 5 days
Precipitation of Wettest Month	CHIRPS	2015-2019	5.4x5.4km^2, 5 days
Precipitation of Driest Month	CHIRPS	2015-2019	5.4x5.4km^2, 5 days
Precipitation Seasonality (Coefficient of Variation)	CHIRPS	2015-2019	5.4x5.4km^2, 5 days
Precipitation of Wettest Quarter	CHIRPS	2015-2019	5.4x5.4km^2, 5 days
Precipitation of Driest Quarter	CHIRPS	2015-2019	5.4x5.4km^2, 5 days
Precipitation of Warmest Quarter	MODIS/CHIRPS	2015-2019	1x1km^2/5.6x5.6km^2, 8 days/5 days
Precipitation of Coldest Quarter	MODIS/CHIRPS	2015-2019	1x1km^2/5.6x5.6km^2, 8 days/5 days

Description	Source	Period	Resolution
Normalised Vegetation Index (annual average)	MODIS	2015-2019	1x1km^2, 15 days
Enhanced Vegetation Index (annual average)	MODIS	2015-2020	1x1km^2, 15 days
Altitude	SRTM DEM		
Proportion of district (admin 2) surface covered by forest (admin 2)	Copernicus Global Land Cover	2019	100x100m^2, annual
Proportion of district (admin 2) surface covered by forest (admin 2)	Copernicus Global Land Cover	2019	100x100m^2, annual
Proportion of district (admin 2) surface covered by crops (admin 2)	Copernicus Global Land Cover	2019	100x100m^2, annual
Proportion of district (admin 2) surface covered by permanent water	Copernicus Global Land Cover	2019	100x100m^2, annual
Proportion of households with improved sanitation (irrespective of shared or not) aggregated at district level	DHS	2014, 2017/18	
Proportion of households with open defecation (aggregated at district level)	DHS	2014, 2017/18	
Proportion of households with improved drinking water sources (aggregated at district level)	DHS	2014, 2017/18	
Proportion of households with handwashing facility (aggregated at district level)	DHS	2014, 2017/18	
Proportion of households with presence of water and soap (aggregated at district level)	DHS	2014, 2017/18	
Population density	facebook	2020	
rural(=1)/urban(=0)	GRUMP/SEDAC	2000	
Koeppen-Geiger climate classification: 120 (Tropical, savannah), 130 (Tropical, monsoon), 321 (Tropical, monsoon), 322 (Temperate, dry winter, warm summer)	Cui et al. (2021)	1988-2017	
Quintiles: 1(poorest),2 (poor), 3 (medium), 4(wealthy), 5 (wealthiest)	DHS	2011	
Bulk density of the fine earth fraction (cg/cm^3)	ISRIC		
Cation Exchange capacity of soil (cmol/kg)	ISRIC		
Volumetric fraction of coarse fragments (>2mm), cm^3/100cm^3, vol%	ISRIC		
Proportion of clay particles (<0.002 mm) in the fine earth fraction (g/100g,%)	ISRIC		
Total nitrogen (cg/kg)			
Organic carbon density (kg/dm^3)	ISRIC		
Organic carbon stocks (kg/dm^3)	ISRIC		
Soil pH (x10)	ISRIC		
Proportion of silt particles ($\geq$ 0.002 mm and $\leq$ 0.05 mm) in the fine earth fraction (g/100g,%)	ISRIC		
Proportion of sand particles (>0.05 mm) in the fine earth fraction (g/100g,%)	ISRIC		

Description	Source	Period	Resolution
Soil organic carbon content in the fine earth fraction (g/kg)	ISRIC		
Admin 0, 1 and 2 (national, division, and district) boundary shapefiles	GADM		

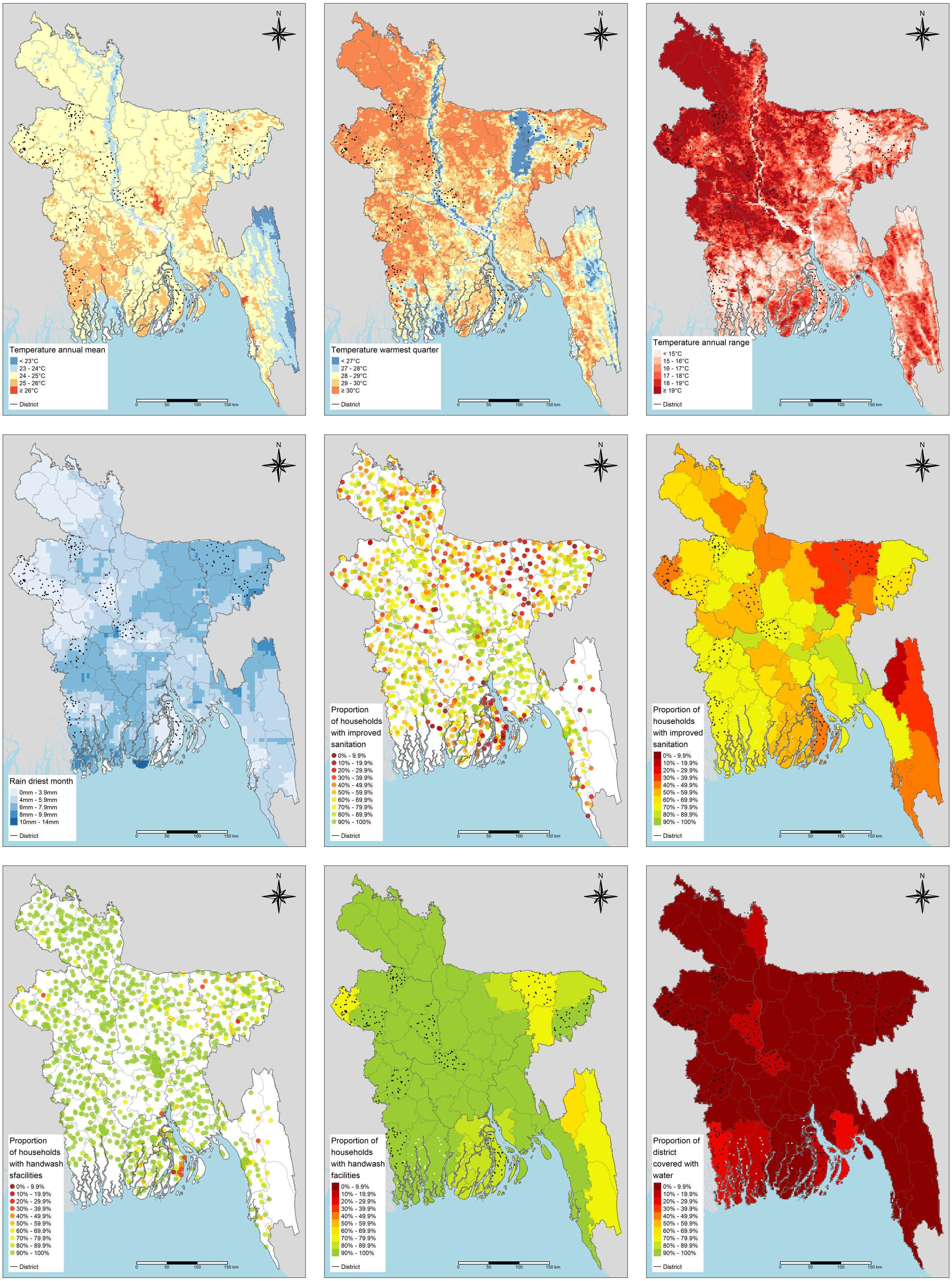

Figure 1: Maps from statistically important covariates based on the bivariate analysis (part 1). These maps were created using R's tmap-package [29] and include administrative boundaries retrieved from gadm.org.

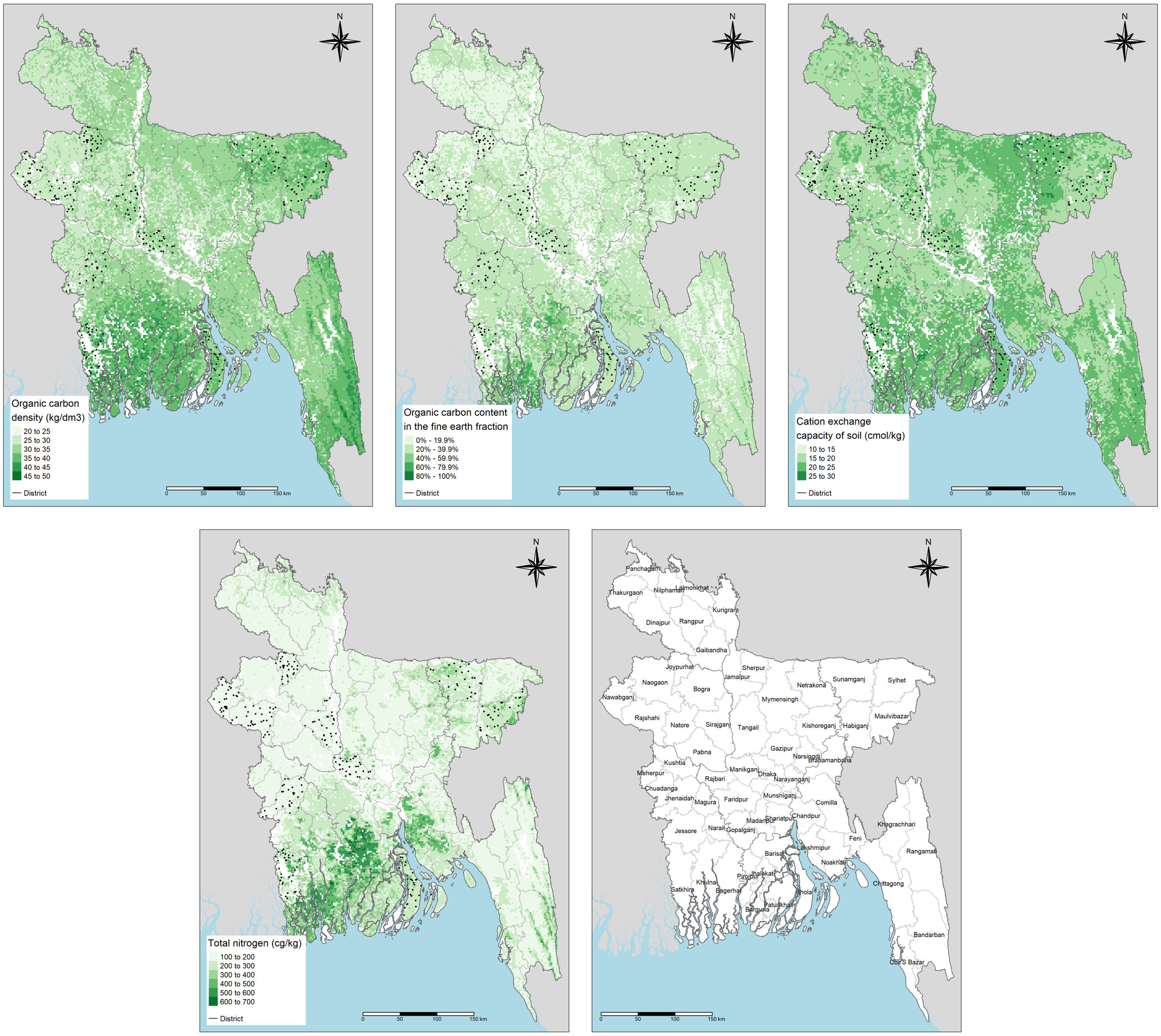

Figure 2: Maps from statistically important covariates based on the bivariate analysis (part 2) and an overview over the 64 districts of Bangladesh. These maps were created using R's tmap-package [29] and include administrative boundaries retrieved from gadm.org.
