# Supplementary material for: Distribution and treatment needs of soil-transmitted helminthiasis in Bangladesh: A Bayesian geostatistical analysis of 2017-2020 national survey data
Source: PLoS Negl Trop Dis. 2023 Nov 6;17(11):e0011656. doi: 10.1371/journal.pntd.0011656 (PMC10662736; doi:10.1371/journal.pntd.0011656)
Supplement: S2 Appendix — (PDF) [file pntd.0011656.s002.pdf]

S2 Appendix: Bayesian geostatistical modeling

Prior to the analysis, the VIF was computed for all predictors at the locations of the observed data and the predictors with the highest VIF, above 10, were removed one at a time. To facilitate the estimation of the model parameters, the continuous covariates were standardized by subtracting and dividing their values by their corresponding mean and standard deviation.

Let Y_i, n_i, and p_i be the number of infected individuals, the number of tested individuals, and the prevalence of infection risk for the respective species at survey location i, respectively. We assumed a binomial distribution for the number of infected individuals $Y_i \sim \text{Binomial}(n_i, p_i)$. The spatial correlation was modeled as a Gaussian process, i.e. as a random effect $\phi_{spatial}$ with $(\phi^{(1)}_{spatial}, \ldots, \phi^{(n)}_{spatial}) \sim \text{MVN}(0, \Sigma)$, such that the spatial correlation is held by the covariance matrix Σ. The spatial correlation is assumed to be stationary, isotropic, and of exponential form, i.e. $\Sigma_{ij} = \sigma^2_{spatial} \cdot \exp(-\rho \cdot d_{ij})$, where the *partial sill* $\sigma^2_{spatial}$ determines the magnitude of the spatial correlation and d_{ij} denotes the distance between two locations. The *decay* parameter ρ incorporates how fast the spatial correlation decays with the distance and is directly linked to the range. Defining the practical range r as the distance at which the spatial correlation drops below $\exp(-2) \approx 13.5$ leads to the relation $r = 2/\rho$ between the practical range and the decay. Additionally, a non-spatial random effect $\phi_{non-spatial} \overset{iid}{\sim} \mathcal{N}(0, \sigma^2_{non-spatial})$ is incorporated to capture local non-spatial noise, which could be due to measurement error or small scale local effects. The magnitude of this non-spatial random effect is measured by the *nugget* $\sigma^2_{non-spatial}$. The predictors, spatial and non-spatial random effects are linked to the prevalence parameter via the logit link function

$$\text{logit}(p_i) = \alpha + X_i^T \beta + \phi_{spatial} + \phi_{non-spatial},$$

where α is the intercept, X a matrix of covariates, and β a vector with one coefficient β_j for each covariate.

For hookworm, the prevalence of infection was 0% in 87.2% of locations. To address the large frequency of zeros in the data, we fitted a zero-inflated binomial model. In short, a zero-inflated binomial model with probability density function

$$f(y_i|\theta, n_i, p_i) = \begin{cases} (1-\theta) \cdot \text{Binomial}(y_i, n_i, p_i) & \text{if } y_i > 0 \\ \theta + (1-\theta) \cdot \text{Binomial}(0, n_i, p_i) & \text{if } y_i = 0 \end{cases}$$

classifies zero prevalence into two categories: zeros which are due to the binomial distribution and structural zeros. These categories are attributed according to a $z \overset{iid}{\sim} \textit{Bernoulli}(\theta)$ distribution, where θ denotes the probability of a structural zero and is called mixing probability. The occurrence of structural zeros can, for example, be attributed to environmental conditions, which may not be favourable for hookworm or observer errors, (see [1] for a more detailed discussion). The probability p_i was linked to the covariates and the spatial and non-spatial random effect via the same link function as the binomial model described above.

To complete the Bayesian formulation, prior distributions for all parameters were specified. In particular, we adopted vague normal priors for the regression coefficients $\beta_j \sim \mathcal{N}(0, 1000)$, an inverse-gamma prior for the non-spatial variance $\sigma^2_{non-spatial} \sim \text{IG}(2.01, 1.01)$, penalized complexity priors [2] for the spatial variance and the range, such that $P(\sigma^2_{spatial} > 4) = 0.01$ and $P(r < 100) = 0.7$, and finally a Gaussian normal prior for the inverse logit of the mixing probability $\text{logit}^{-1}(\theta) \sim \mathcal{N}(-1, .02)$ of the zero inflated model.

References

[1] Zuur AF, Ieno EN. Beginner's guide to spatial, temporal and spatial-temporal ecological data analysis with R-INLA: GAM and zero-inflated models. Newburgh, UK: Highland Statistics Ltd; 2018.

[2] Fuglstad G-A, Simpson D, Lindgren F, Rue H. Constructing priors that penalize the complexity of Gaussian random fields. J Am Stat Assoc. 2019;114: 445–452. doi:10.1080/01621459.2017.1415907
