# Supplementary material for: Distribution and treatment needs of soil-transmitted helminthiasis in Bangladesh: A Bayesian geostatistical analysis of 2017-2020 national survey data
Source: PLoS Negl Trop Dis. 2023 Nov 6;17(11):e0011656. doi: 10.1371/journal.pntd.0011656 (PMC10662736; doi:10.1371/journal.pntd.0011656)
Supplement: S3 Appendix — (PDF) [file pntd.0011656.s003.pdf]

S3 Appendix: Model estimates including risk groups

Table 1 and 2 display the estimates of the geostatistical model used for predicting the treatment needs for PSAC, SAC, and adults respectively WRA.

Table 1: Median (95%-BCI) estimates of the parameters posterior distributions of the geostatistical model for the prevalence of each STH species including age. The parameters for the regression coefficients are given as odds ratios.

	A. lumbricoides	*T. trichiura*	Hookworm
Temp: Annual mean		0.89 (0.59; 1.22)	
Temp: Annual range	0.85 (0.61; 1.11)		
Temp: Mean warmest quarter	0.96 (0.74; 1.18)	0.82 (0.56; 1.11)	
Rain: Driest month	0.69 (0.48; 0.90)		
Soil: Cation exchange capacity			0.88 (0.49; 1.32)
Soil: Organic carbon density	1.37 (1.05; 1.71)		1.18 (0.48; 2.02)
Soil: Nitrogen			1.05 (0.28; 1.99)
Soil: Organic carbon (%)			0.32 (0.05; 0.65)
Wash: Improved sanitation	0.25 (0.09; 0.46)	0.36 (0.15; 0.62)	
Wash: Handwashing facility	2.31 (0.85; 4.01)		
Landcover: Water (%)			0.51 (0.10; 1.00)
Age:			
PSAC	1	1	1
SAC	1.10 (0.89; 1.32)	1.16 (0.85; 1.48)	1.76 (0.61; 3.22)
Adults	1.03 (0.83; 1.24)	0.97 (0.70; 1.26)	1.80 (0.62; 3.31)
$\sigma^2_{non-spatial}$	0.23 (0.14; 0.35)	0.19 (0.10; 0.33)	0.48 (0.16; 1.15)
$\sigma^2_{spatial}$	0.85 (0.41; 1.56)	2.26 (1.11; 4.12)	0.53 (0.03; 1.87)
Range (km)	81 (38; 148)	88 (47; 151)	127 (5; 593)
Mixing probability θ			0.10 (0.00; 0.39)

Table 2: Median (95%-BCI) estimates of the parameters posterior distributions of the geostatistical model for the prevalence of each STH species including WRA. The parameters for the regression coefficients are given as odds ratios.

	A. lumbricoides	*T. trichiura*	Hookworm
Temp: Annual mean		0.88 (0.58; 1.20)	
Temp: Annual range	0.85 (0.60; 1.10)		
Temp: Mean warmest quarter	0.96 (0.75; 1.19)	0.87 (0.59; 1.17)	
Rain: Driest month	0.66 (0.46; 0.88)		
Soil: Cation exchange capacity			0.89 (0.50; 1.33)
Soil: Organic carbon density	1.34 (1.02; 1.68)		1.18 (0.48; 2.00)
Soil: Nitrogen			1.06 (0.28; 2.00)
Soil: Organic carbon (%)			0.32 (0.05; 0.66)
Wash: Improved sanitation	0.26 (0.10; 0.48)	0.38 (0.16; 0.65)	
Wash: Handwashing facility	2.30 (0.84; 3.98)		
Landcover: Water (%)			0.51 (0.11; 0.99)
WRA:			
No	1	1	1
Yes	1.06 (0.86; 1.27)	0.89 (0.64; 1.16)	1.40 (0.59; 2.33)
$\sigma^2_{non-spatial}$	0.24 (0.15; 0.36)	0.28 (0.15; 0.48)	0.44 (0.16; 1.02)
$\sigma^2_{spatial}$	0.86 (0.37; 1.77)	2.46 (1.10; 4.99)	0.37 (0.05; 1.02)
Range (km)	103 (49; 199)	120 (60; 228)	88 (9; 326)
Mixing probability θ			0.07 (0.00; 0.23)
