# Supplementary material for: Distribution and treatment needs of soil-transmitted helminthiasis in Bangladesh: A Bayesian geostatistical analysis of 2017-2020 national survey data
Source: PLoS Negl Trop Dis. 2023 Nov 6;17(11):e0011656. doi: 10.1371/journal.pntd.0011656 (PMC10662736; doi:10.1371/journal.pntd.0011656)
Supplement: Attachment — Submitted filename: Gerber et al. Response letter.pdf [file pntd.0011656.s005.pdf]

We are very thankful to all three reviewers for the positive evaluation of our work, along with helpful comments and suggestions for language corrections to further improve quality and clarity of our manuscript. Below, please find our point-by-point response, clearly indicating how and where in the manuscript changes have been done.

1 Reviewer #1

Thank you very much for taking the time to thoroughly review our manuscript. Your comments and questions were particularly helpful to challenge our reasoning once more and to force us to state our arguments more clearly. We respond to your comments point by point (Reviewer comment in black, author comment in blue, quotations from the manuscript are inline, changes to the manuscript are inline and highlighted with yellow).

1.1 Summary

This is an interesting paper looking at a relevant, topical area. How can we measure the impact of STH programs. How can programs adjust their approach in light of new WHO treatment guidance. And how can geostatistical tools be helpful in that regard?

1.2 Comments

Is predicting the prevalence over a 1km x 1 km high resolution enough? STH is less focal than other diseases (such as schistosomiasis for example) of course but is greater granularity required?

This is a very interesting point. As you mention, STH is not as focal than for example schistosomiasis. This is also reflected in the estimated range of above 100km for all STH species. We therefore pragmatically chose the resolution as small as possible, but such that the computations would still be feasible in reasonable time: The lower bound of the chosen resolution was driven by the available computation time. To predict at 1km x 1km resolution and especially to process and aggregate the points over the shapes of different administrative boundaries, took days on our high performance computers (40 cores parallel processing). Reducing the resolution by half, i.e. to 0.5km x 0.5km, would increase the number of points and hence the computation time by a factor 4. Given that most of the covariates were not available at a resolution smaller than 1km x 1km, we argue that we could not gain much more precision by taking a smaller resolution.

Line 18-19 – references a national treatment coverage of 98.3%. Is that for one round of MDA or a cumulative figure? That is impressively high.

The referenced sentence in the submitted manuscript was:

> Ongoing, this deworming program had reached a national treatment coverage increasing from 86.4% to 98.3% in the five year period 2014-2019 and has distributed a total of 78.2 mio. tablets in 2019 alone [9].

In the referenced database, Bangladesh is listed with the following figures:

country	year	Reported number of SAC treated	Drug combination, SAC	National coverage, SAC (%)
Bangladesh	2014	600'168	Dec+Alb	1.89
Bangladesh	2014	26'429'210	Mbd - round 1	83.34
Bangladesh	2014	27'789'903	Mbd - round 2	87.63
Bangladesh	2015	476'818	Dec+Alb	86.40
Bangladesh	2015	26'704'597	Mbd - round 1	86.40
Bangladesh	2015	26'600'439	Mbd - round 2	86.40

Bangladesh	2016	26’396’187	Mbd - round 1	86.37
Bangladesh	2016	26’834’605	Mbd - round 2	86.37
Bangladesh	2017	37’752’460	Mbd - round 1	95.36
Bangladesh	2017	38’304’656	Mbd - round 2	95.36
Bangladesh	2018	38’920’591	Mbd - round 1	98.25
Bangladesh	2018	38’703’636	Mbd - round 2	98.25
Bangladesh	2019	39’228’037	Mbd - round 1	98.27
Bangladesh	2019	38’955’227	Mbd - round 2	98.27

We realized that the number in the manuscript corresponds to the national coverage in 2015 rather than 2014. in 2015, the reported national coverage was are the same (86,4%) for a small Dec+Alb distribution and for the two Mbd rounds. The corresponding line in the manuscript was changed to:

> Ongoing, this deworming program had reached a national treatment coverage increasing from 86.4% to 98.3% in the four year period 2015-2019 and has distributed a total of 78.2 mio. tablets in 2019 alone [9].

Line 29-30 and 69-70 – why were these 10 districts chosen? Were they done so with sample size calculations underpinning them, or was it from a convenience sampling approach? How comfortable are you making predictions for the South East region of the country where there are no actual samples?

To answer the first part of the question, our study is a secondary analysis relying on data collected and published by Dhakal et al. 2020. Dhakal et al. state that the sampling districts were selected by the Bangladesh ELFSTH according to programmatic priorities. We refer to Dhakal et al. for more details on the programmatic priorities.

To answer the second part of the question, as mentioned in the discussion section lines 304 – 317, we are cautioning against using the predictions for the northeastern and the southwestern part of Bangladesh in the light of the absence of collected data in these regions. We also recommend to collect more data in these regions to be able to make more robust predictions of the prevalence in these regions.

Line 33 – are these baseline figures comparing the same areas as those in the current study?

The reported numbers are national figures. To the best of our knowledge, no district prevalence figures are available for this time period. To make this point more clear, we changed the sentence

> Indeed, in 2003, the prevalence of A. lumbricoides, T. trichiura, and hookworm in the population was 55.0%, 46.0%, and 35.0%, respectively.

to

> Indeed, in 2003, the prevalence of A. lumbricoides, T. trichiura, and hookworm in the population of Bangladesh was 55.0%, 46.0%, and 35.0%, respectively.

The estimates on the potential reduction in treatment are interesting. In the authors’ opinion is the BGM output alone enough to determine treatment approach country wide? Or do they need to be supplemented with IU/EU specific surveys required?

Like every method, BGM has advantages and disadvantages. We therefore advocate that programmatic decisions are always made based on plurality of methods. The hope is, that the disadvantage of various methods will cancel out. However, the advantages of BGM (e.g. spatial extrapolation of information at very low cost) can help to inform the decision making.

Model validation, lines 116-117. ‘Model validation was performed by fitting each model to a randomly selected subset of 80% locations (training set) and predicting the disease risk at the remaining ones (testing set).’ Does that mean taking 80% of the ten districts, or 80% of the 296 villages? Or another approach?

It means 80% of the 296 villages. To make this more clear, we changed the sentence from

> Model validation was performed by fitting each model to a randomly selected subset of 80% locations (training set) and predicting the disease risk at the remaining ones (testing set).

to

> Model validation was performed by fitting each model to a randomly selected subset of 80% of the community locations (training set) and predicting the disease risk at the remaining ones (testing set).

Lines 176 – 177. Socioeconomic factors were not significant. Isn’t that surprising?

Socioeconomic factors are undoubtedly a relevant driver for STH infection at an individual level (e.g. wearing of closed shoes) or even at household level (e.g. sealed floor). In our study, however, prevalence and the socioeconomic proxy was aggregated at community level. This means that individuals with and without shoes and households with sealed floor or not were analyzed at the same time. Our statement merely indicates that socioeconomic factors are not suited to estimate STH prevalence at the community level.

Lines 178-184 – this seems to repeat the information in the paragraph above. Am I confusing something?

Lines 162- 177 refer to the variable selection process called bivariate geostatistical analysis. For this the prevalence was explained by one covariate at a time. Lines 178-184 refer to the model estimation of the final model, which includes all statistically relevant variables uncovered from the bivariate geostatistical analysis. We have changed the two paragraph beginnings to make this more clear from

> The results from the bivariate geostatistical analysis in Figure 2 suggest that […]
>
> […]
>
> Estimates of the model fits are displayed in Table 2. […]

to

> The results from the variable selection process through bivariate geostatistical analysis in Figure 2 suggest that […]
>
> […]
>
> Models including all variables from the variable selection process were fitted and their parameter estimates displayed in Table 2. […]

Table 2 – is it possible to calculate for the combined STH species? That's what determines treatment approach.

The population-adjusted prevalence for the combined STH species are displayed in line 4 under the row heading 'Any STH'.

Lines 223-224 – are the numbers for WRA contained within the adults number or are they separate?

WRA are contained within the adults number. To make this more clear, we have adjusted the lines 223-225 from

> […] and 6.2 million (5; 8) WRA were infected with any STH. No statistically important prevalence differences between risk groups could be observed.

to

> […] and 6.2 million (5; 8) WRA were infected with any STH. WRA were considered a subset of adults. No statistically important prevalence differences between risk groups could be observed.

Minor / Editorial

Line 2 – 'infection' should probably be 'infections'

Line 279 – 'patters' should probably be 'patterns'

Line 292 – 'trice' should probably be 'thrice'

Thank you very much for reading the manuscript so carefully and to suggest these orthographical improvements. We have changed the corresponding lines to:

Line 2 - If untreated, STH infections can lead to intestinal obstruction, abdominal pain or bleeding, anemia, and malnourishment [2, 3].
Line 279 - The detection of such local prevalence patterns allows for a more targeted allocation of treatments.
Line 292 This would imply that treatment would be stopped in two districts, while it would be continued in 30, 11, 20, and 1 districts at a frequency of every 2 years, yearly, twice a year, and thrice a year, respectively, requiring a total of 32.0 million tablets annually for SAC.
Line 296 - Furthermore, using the more conservative upper 95% BCI bound of the district prevalence estimate for a treatment decision, would imply that treatment would be continued in 16, 12, 25, and 11 districts at a frequency of every two years, yearly, twice a year, and thrice a year, respectively, requiring a total of 48.8 million tablets annually for SAC.

2 REVIEWER #2

Thank you very much for taking the time to thoroughly review our manuscript. Your comments and questions were particularly helpful to challenge our reasoning once more and to force us to state our arguments more clearly. We respond to your comments point by point (Reviewer comment in black, author comment in blue, quotations from the manuscript are inline, changes to the manuscript are inline and highlighted with yellow).

2.1 SUMMARY

This paper uses Bayesian geostatistical models and spatially indexed environmental data to predict STH prevalence nationwide using data from 10 out of 64 districts. This study's findings are useful for informing whether MDA should be focally targeted and/or modified in future years. However, there are some limitations of the analysis that have not been adequately addressed. In addition, more detail is needed about the data processing to understand the risk of misclassification of the risk factors.

2.2 COMMENTS

2.2.1 Methods

1. There is not sufficient detail about how the risk factor data was matched to Kato-Katz data with respect to space and time. Authors mention that DHS data was aggregated to district level. Were the environmental risk factors at their original level of resolution matched to individual STH data? The time period and time resolution of the risk factors is mentioned, but it is not clear whether the matching to outcome data accounted for the possibility of a long lag time between exposure and STH measurement, especially because some STH infections can last for months.

Thank you very much for pointing us to this unclear formulation. We added the clarification

> In this study, we defined the infection status of an individual according to the maximum egg count of the duplicate Kato-Katz thick smears. The individual infection status was aggregated at the community level to estimate the prevalence in a community. The unit of analysis was this community prevalence, which was georeferenced according to the centroid of the surveyed households within a community.

And changed

> The aforementioned predictors were extracted or derived at the disease survey locations.

To

> The aforementioned predictors were extracted or derived at the community locations.

2. The unit of analysis is not clear. S2 Appendix mentions that the model was for prevalence by "survey location". Is this district, village, or something else? This is important for understanding the purpose and appropriateness of random effects and should be mentioned at least briefly in the main text.

Together with the change above, which includes a statement of the unit of analysis, we clarified this point by explicitly restating the unit of analysis in the text of the statistical methods. For this, we changed

> Line 107 - To address collinearity, we excluded covariates with a variance inflation factor above 10 [23]. For modeling the prevalence, species-specific Bayesian geostatistical logistic regression models with spatially structured and independent random effects were developed [24]. Details […]

to

> Line 107 - To address collinearity, we excluded covariates with a variance inflation factor above 10 [23]. For modeling the prevalence, species-specific Bayesian geostatistical logistic regression models with spatially structured and independent random effects were developed [24]. The unit of analysis was the community level prevalence. Details […]

and

> Line 116 - Model validation was performed by fitting each model to a randomly selected subset of 80% locations (training set) and predicting the disease risk at the remaining ones (testing set).

to

> Line 116 - Model validation was performed by fitting each model to a randomly selected subset of 80% of the community locations (training set) and predicting the disease risk at the remaining ones (testing set).

3. In the methods, it would be helpful to include more detail and a citation for the Facebook population density data.

The facebook population data is generated as follows: 1. Census data for the area of interest is obtained. 2. Assuming that people live there must be housing, an algorithm retrieves building shapes from satellite images. 3. The area of interest is divided into small tiles and the building density per tile is calculated. 4. The total population is distributed across the area of interest according to the building density per tile.

> Line 103 - Finally, all calculations involving population relied on facebook population density data for 2020 at 100 x 100 m resolution .

to

> Line 103 - Finally, all calculations involving population relied on meta (previously facebook) population density data for 2020 at 100 x 100 m resolution [Reference to facebook data]. This population density data is generated by combining census data with the number of buildings found on high resolution satellite images.

4. In Figure 2, it is not clear from the labels or caption whether these are results from bivariate models or whether models were adjusted for other covariates simultaneously. It would be preferable to adjust for other covariates in each risk factor's model.

The bivariate analysis for which we use a single risk factor in turn to explain the prevalence serves as variable selection process. The output of this bivariate analysis is displayed in Figure 2. Once risk factor candidates are selected in this way, they are included simultaneously in the main analysis. The output of the main analysis is displayed in Table 2. To make this more clear, we have changed the two paragraph beginnings from

> The results from the bivariate geostatistical analysis in Figure 2 suggest that […]
>
> […]
>
> Estimates of the model fits are displayed in Table 2. […]

to

> The results from variable selection process through bivariate geostatistical analysis in Figure 2 suggest that […]
>
> […]
>
> Models including all variables from the variable selection process were fitted and their parameter estimates displayed in Table 2. […]

Additionally, we have adapted the captions of Figure 2 and Table 2 to make the difference even more clear from:

> Caption Figure 2: Estimates (posterior mean and 95 % BCI) of the effects of the risk factors obtained from STH species-specific, bivariate, geostatistical regression models.
>
> Caption Table 2: Parameter estimates (posterior median and 95% BCI) of the geostatistical models for each STH species.

to

> Caption Figure 2: Estimates (posterior mean and 95 % BCI) of the effects of the risk factors obtained from STH species-specific, bivariate, geostatistical regression models used for variable selection purposes.
>
> Caption Table 2: Parameter estimates (posterior median and 95% BCI) of the final multivariable geostatistical models for each STH species.

2.2.2 Results

1. It wasn't clear to me why some of the variables from Fig 2 are not included in Table 2.

Please, see explanation under 2.2.1 Methods, Point 4.

2. Uncertainty estimates from the third column of Figure 1 should be discussed in more detail in the Results and Discussion section. Also, is uncertainty from the models presented in Figure 1 carried forward in the results presented in table 3? If not, this should be mentioned as a limitation.

The uncertainty presented in Figure 1 carry forward to the results in Table 3 and is reflected in the 95%-Bayesian credible intervals. To make this more clear the caption of Table 3 was modified from

> Population-adjusted prevalence by STH species, total number of people and number of persons living in areas with different endemicity and annualized treatment needs by program strategy, stratified by risk group.

to

> Population-adjusted prevalence by STH species, total number of people and number of persons living in areas with different endemicity and annualized treatment needs by program strategy, stratified by risk group. The reported numbers are absolute values or the median and 95\%-BCI of the posterior predictive distribution.

2.2.3 Conclusions

1. A limitation of the study that is not mentioned is that Kato-Katz likely has low sensitivity, especially in a setting where there have been many years of MDA. Prior work in Bangladesh comparing Kato-Katz and qPCR found false negatives as well as false positives for Ascaris. At minimum, the authors should address this limitation and discuss how it could influence their findings. If the prevalence data used to fit models is under-estimated, then the estimates of necessary doses of anthelminthic treatment are likely also under-estimated. https://journals.plos.org/plosntds/article?id=10.1371/journal.pntd.0008087

Thank you for spotting this interesting point. We included this in the discussion by adding the following:

Economically, while assuming a price of US$ 0.25 per treatment, these two treatment decisions would reduce the annual treatment costs for SAC by US$ 11.5 million and US$ 7.4 million, respectively. However, this result has to be taken with a grain of salt because another study, which was conducted in rural Bangladesh, found that Kato-Katz sampling leads to an overestimation of the prevalence of A. lumbricoides and an underestimation of the prevalence of T. trichiura and hookworm [Reference]. It is likely that our estimation data suffers from the same diagnostic uncertainties, which might lead to an overestimation or underestimation of the treatment needs. However, it is also possible, that the overestimation of A. lumbricoides and the underestimation of the prevalence of the other STH species cancel out.

2. The Discussion section on associations between sanitation and STH should also discuss the results of a randomized trial of water and sanitation and hygiene interventions in Bangladesh on STH infections and whether these align with associations in the study. https://www.ncbi.nlm.nih.gov/pmc/articles/PMC6519840/

Thank you for bringing in this aspect to the discussion. We have included the point in the discussion section like so:

For T. trichiura, the only identified risk factor was the proportion of households with improved sanitation, which was negatively associated with infection prevalence. As for A. lumbricoides, this finding is widely established in the literature [30] and was recently reaffirmed recently for T. trichiura [Reference].

3. In the Discussion, when talking about overtreatment, it seems important to mention increasing concerns about anthelminthic resistance.

This was addressed by adding the following sentence to the discussion:

Economically, while assuming a price of US$ 0.25 per treatment, these two treatment decisions would reduce the annual treatment costs for SAC by US$ 11.5 million and US$ 7.4 million, respectively. Beyond the economic gains, the outlined reduction in overtreatment would also counter the growing concern of anthelmintic drug resistance.

2.2.4 Editorial and Data Presentation Modifications?

1. In the Abstract “open-access, model-based databases” is vague. I suggest rephrasing and considering adding one sentence to the abstract methods on the data used.

2. In the concluding sentence of the Abstract, it should state that prevalence is estimated or model-based prevalence.

It is true that the description of some data sources is vague in the abstract. However, due to the editorial limitations of 300 words of which we have used up 299, it is not possible to accommodate a further sentence with more details on the data. We hope that the interested reader will read the details on the data in the Methods section.

To clarify further that the prevalence is always an estimated one, we changed

> There were 24 out of 64 districts with population-adjusted STH prevalence above 20%.

to

> There were 24 out of 64 districts with an estimated population-adjusted STH prevalence above 20%.

3. Line 15: it would be helpful to clarify the ages of children treated in the national deworming program.

The targeted group of SAC is elementary school children 1-5 classes or 5-12 age-group at the time when the report was written (2011). It also stated that a challenge was to also include the 13-14 years age-groups in the MDA program because they were not in elementary school anymore. It remains unclear, whether this challenge was overcome. To clarify this, we changed

> In line with the WHO recommendations, Bangladesh adopted the goal of eliminating STH infection as a public health problem and initiated a national deworming program based on school mass drug administration (MDA) at six-month intervals, which started in 2008 [8].

to

> In line with the WHO recommendations, Bangladesh adopted the goal of eliminating STH infection as a public health problem and initiated a national deworming program based on school mass drug administration (MDA) at six-month intervals at elementary schools (age-groups 6-12), which started in 2008 [8].

However, the aim our our study was not to compare STH prevalence among school attending versus non attending children. This issue was discussed by Dhakal et al. (2020).

4. In Appendix S1 Figures 1-2, it is not clear which time period the data was subset to or whether it was aggregated over time.

To clarify this, we changed the caption of S1 Appendix, Table 1 from

> Description, source and resolution of the covariates included in the analysis.

to

> Description, source, time period of collection and spatial and time resolution of the covariates included in the analysis. If the period of collection contained several measurements, the data was aggregated over the indicated time period.

5. Figure 1 image resolution is too low to read the legends and to see the results clearly.

This point will be clarified with the editors. The uploaded Figures are at a higher resolution (such that labels are easily readable) and we suspect that the pdf rendering of the submission document reduced the resolution.

6. Since DHS data was aggregated to district level, in the results, the text and figures/tables should be revised to clarify this. As written, it could imply household-level sanitation, for example.

The aggregation of the DHS data is mentioned in the methods. However, to avoid confusion, we have added a footnote to Table 2:

> Aggregated at district level.

We further changed the text of the results in the following way:

Line 178 - The main predictors of A. lumbricoides prevalence were the proportion of households with improved sanitation (statistically important negative effect) and the amount of rainfall in the driest month of the year (negative effect). The proportion of households with improved sanitation was the only statistically important predictor of T. trichiura prevalence, indicating that the higher the proportion, the lower the prevalence.

to

Line 178 - The main predictors of A. lumbricoides prevalence were the proportion of households per district with improved sanitation (statistically important negative effect) and the amount of rainfall in the driest month of the year (negative effect). The proportion of households per district with improved sanitation was the only statistically important predictor of T. trichiura prevalence, indicating that the higher the proportion, the lower the prevalence.

From

Line 162 - The results from the bivariate geostatistical analysis in Figure 2 suggest that the factors with a statistically important protective effect (in statistical terms) on A. lumbricoides prevalence were the proportion of households with improved sanitation and hand washing facilities, the mean temperature of the warmest quarter, the temperature annual range, and the precipitation of the driest month of the year.

to

Line 162 - The results from the bivariate geostatistical analysis in Figure 2 suggest that the factors with a statistically important protective effect (in statistical terms) on A. lumbricoides prevalence were the proportion of households per district with improved sanitation and hand washing facilities, the mean temperature of the warmest quarter, the temperature annual range, and the precipitation of the driest month of the year.

From

Multiple geostatistical logistic regression analysis across the whole population showed that on one hand, the higher the proportion of households with improved sanitation, the lower the prevalence of A. lumbricoides. This is an established association [28] and a factor which is taken into account by WHO's control strategy [29]. On the other hand, the higher the precipitation in the driest month of the year, the lower the prevalence of this species. This risk factor might be associated with the parasite's reproduction cycle or human behavior. For T. trichiura, the only identified risk factor was the proportion of households with improved sanitation, which was negatively associated with infection prevalence.

to

Multiple geostatistical logistic regression analysis across the whole population showed that on one hand, the higher the proportion of households per district with improved sanitation, the lower the prevalence of A. lumbricoides. This is an established association [28] and a factor which is taken into account by WHO's control strategy [29]. On the other hand, the higher the precipitation in the driest month of the year, the lower the prevalence of this species. This risk factor might be associated with the parasite's reproduction cycle or human behavior. For T. trichiura, the only identified risk factor was the proportion of households per district with improved sanitation, which was negatively associated with infection prevalence.

3 Reviewer #3

Thank you very much for taking the time to thoroughly review our manuscript. Your comments and questions were particularly helpful to challenge our reasoning once more and to force us to state our arguments more clearly. We respond to your comments point by point (Reviewer comment in black, author comment in blue, quotations from the manuscript are inline, changes to the manuscript are inline and highlighted with yellow).

3.1 Summary

This is an informative and important analysis. One major limitation is that STH data are only available from 10 out of 64 districts. The authors acknowledge this in the discussion section but this information should also be included in the abstract and in the methods sections under data.

To accommodate this limitation in the abstract, we changed the sentence

> To evaluate the success of this strategy, surveys were conducted between 2017 and 2020 in 10 districts.

in the Background section to

> To evaluate the success of this strategy, surveys were conducted between 2017 and 2020 in 10 out of 64 districts.

3.2 Comments

3.2.1 Methods

The methods are robust. Line 192: Can the authors explain what a high mixing probability is and what structural zeros are?

Mixing probability and structural zeros are terms used in the context of zero inflated modeling. Technically, zero inflated models are a combination of a process, which determines if a location is eligible for a non-zero value and a process, which determines the count if the non-zero value is eligible. Locations not eligible for non-zero values are called structural zeros, the others random zeros. One may think of the structural zeros as a part of the Bangladesh, which is unsuitable for STH transmission, e.g. mountain caps or areas with high usage of MDA such that hookworm cannot thrive. The mixing proportion corresponds to the estimated proportion of structural zeros. A high mixing proportion means that the probability for structural zeros was high.

3.2.2 Results

The results are clearly presented.

3.2.3 Conclusions

Line 249: Please elaborate on how the parasites reproduction cycle or human behavior are expected to to explain the observed association with precipitation. What is the evidence from previous studies on the relationship between precipitation and Ascaris?

While writing the corresponding lines, we suspected an association between the eggs survival time and the humidity. However, closely inspecting the argument again, we found no literature supporting or contradicting precipitation in the wettest month as a protective risk factor. Our suspicion was further that people would be less outside and have fewer contacts with other people if there was more precipitation, which may reduce the risk of egg ingestion. We could however also not find any supporting or contradicting evidence for this argument. To flag this lack of support of the argument in the literature, we changed

> This risk factor might be associated with the parasite's reproduction cycle or human behavior.

To

> We can only speculate that more precipitation in the driest month leads to a smaller chance of survival for the eggs outside the host or that more precipitations leads to a change in human behavior, e.g. the reduction of contacts, with might be decreasing transmission.

Lines 255-261: Can the authors compare and contrast the treatment coverage in Indonesia and South Africa to Bangladesh to substantiate the statement that differences in dominant risk factors may be driven by differences in treatment coverage?

Our reasoning is that a risk factor might be associated with the prevalence of a STH species through a known or unknown causal relationship. While this causal relationship may be apparent in situations with no or little mass drug administration (like in South Africa with no reported treatment), it may be reduced to no more than a random

association in situation where the prevalence has been heavily influenced by a third variable – the mass drug administration. We have clarified our reasoning by changing:

> This is in line with previous research [31], that reported found a positive association with hookworm prevalence compared to Strongyloides stercoralis prevalence in Indonesia, but contradicts another study [32], that found a negative association for South Africa.
>
> Comparisons of risk factors to situations in other time periods or geographical areas may be limited by the consistently high treatment coverage in Bangladesh, which may have weakened the statistical relation between risk factors and STH prevalence.

to

> This is in line with previous research [31], that reported found a positive association with hookworm prevalence compared to Strongyloides stercoralis prevalence in Indonesia, but contradicts another study [32], that found a negative association for South Africa.
>
> Comparisons of risk factors to situations in other time periods or geographical areas may be limited by different collinearity structures among the covariates [Dormann et al.] or different levels of treatment coverage: While a causal relationship would be reflected in a statistical association in a situation with no mass drug administration like South Africa in 2004, the same causal relationship could be no more than random noise in a situation where the prevalence has been heavily influenced by a third factor like the consistently high national treatment coverage of MDA in Bangladesh of above 80% since 2013 [Reference to PCT databank].
